# Clinical characteristics and improvement of the guideline-based management of acute myocardial infarction in China: a national retrospective analysis

**DOI:** 10.18632/oncotarget.14890

**Published:** 2017-01-29

**Authors:** Lechen Wang, You Zhou, Cheng Qian, Yanggan Wang

**Affiliations:** ^1^ Department of Cardiology, Zhongnan Hospital of Wuhan University & the Medical Research Institute of Wuhan University, Wuhan University, Wuhan, China

**Keywords:** acute myocardial infarction, quality of care, epidemiology, China

## Abstract

**Objective:**

This study is to document the clinical characteristics and improvement in management of acute myocardial infarction (AMI) in Chinese population.

**Results:**

This study included 64,654 patients (23,805 patients in 2011, 40,849 patients in 2013), of which STEMI and NSTEMI account for 85.09% and 14.91%, respectively. From 2011 to 2013, significant improvement has been achieved in the recanalization rate of PCI (96.01% *vs*. 98.63%, *P* < 0.001) and in-hospital deaths (*4.52% vs. 3.55%*, *P* = 0.038). Although the time of door-to-balloon and the duration of PCI were satisfactorily controlled within 90min and 60min, respectively, the onset-to-FMC time (≈3.5h) and door-to-thrombolysis time (≈1.1h) limited the efficiency of management. The total cost of medical care showed no increase from 2011 to 2013, but the patient's paid Portion decreased from 20.33% to 13.96%.

**Materials and Methods:**

The AMI patients admitted in the general hospitals in 2011 and 2013 were retrospectively analyzed according to the data reported to the Single Disease Quality Control Information Systemissued by Chinese Hospital Association.

**Conclusion:**

Compared to the Western countries, STEMI accounted for a larger portion of AMI, and the AMI management in China basically meets the standards of the quality control of guidelines. With improvement of management, there was no increase in the total medical cost, while the patient's paid portion was actually reduced. In future, improvement of transportation strategy and the public medical education are recommended to shorten the onset-to-FMC time to further improve the outcome of AMI patients.

## INTRODUCTION

Contrast to the Western society, the incidence of acute myocardial infarction (AMI) increases dramatically in China with the change of lifestyle and prolonged life span. Currently, there are approximately 2.5 million AMI patients in China [1], and this figure is predicted to approach 23 million in 2030 [2]. According to the Health Statistics issued by the National Health and Family Planning Commission in 2012 [3], AMI is the leading cause of death which accounts for 7.64% and 7.60% of the total deaths in Chinese urban and rural areas in 2011, respectively. To our knowledge, no studies have characterized the current clinical characteristics and medical management of AMI in Chinese population. The present study aims to assess the clinical profiles and the improvement in management of AMI in Chinese population in 2011 and 2013.

## RESULTS

### General characteristics of the patients

Overall, in 2011 and 2013, 64,654 patients were included in this study, of which STEMI and NSTEMI account for 85.09% and 14.91%, respectively. The average age at the onset of STEMI was 62.5 ± 12.8 years old, with the youngest age of 24 and the oldest age of 94, while the average age for the patients with NSTEMI was 65.5 ± 12.6, about 3 years older than the STEMI patients (*P* < 0.001). The peak age for STEMI and NSTEMI was 50-79 years old, accounting for 73.08% and 74.24%, respectively. According to the results of angiography, for both STEMI and NSTEMI, the left anterior descending (LAD) artery was the most commonly affected vessel (≈54%), followed by the right coronary artery and circumflex artery (28% and 17%, respectively), while only 0.5% of patients had the left main artery involvement. Compared to NSTEMI, more patients with STEMI had undergone emergency PCI (51.00% *vs*. 21.15%, *P* < 0.001) with higher rate of complete occlusion of coronary arteries (58.20% *vs*. 38.46%, *P* < 0.001) (Table 1).

**Table 1:** Characteristics of patients with STEMI and NSTEMI

Indicators	STEMI (n=55,014)	NSTEMI (n=9,640)	*P* value
**Age, years**	62.5±12.8	65.5±12.6	<0.001
**Peak age at AMI, years (%)**	50-79 (73.08)	50-79 (74.24)	0.32
**Lesion location, n (%)**			
**Anterior wall**	27002 (49.08)	1753 (18.18)	<0.001
**Inferior wall**	22225 (40.40)	1560 (16.18)	<0.001
**Unspecified-site**	5787 (10.52)	6327 (65.63)	<0.001
**Affected vessels, n (%)**			
**Left anterior descending artery**	29763 (54.10)	5190 (53.84)	0.634
**Right coronary artery**	15338 (27.88)	2765 (28.68)	0.107
**Circumflex artery**	9589 (17.43)	1646 (17.07)	0.406
**Left main artery**	324 (0.59)	39 (0.40)	0.029
**Emergency PCI, n (%)**	28057 (51.00)	2039 (21.15)	<0.001
**Vascular occlusion, n (%)**	32018 (58.20)	3708 (38.46)	<0.001
**Ejection fraction, %**	53.8±10.9	55.1±10.6	<0.001
**Left ventricular diameter, mm**	49.8±6.2	50.0±6.5	0.79
**Pulmonary hypertension, n (%)**	3774 (6.86)	925 (9.60)	<0.001

AMI, acute myocardial infarction; NSTEMI, non ST-segment elevation myocardial infarction; PCI, percutaneous coronary intervention; STEMI, ST-segment elevation myocardial infarction.

Patients were divided into six groups by age (years): < 40, 40-49, 50-59, 60-69, 70-79, ≥80. The types and location of MI and the affected arteries were then analyzed and the results were summarized in Table 2. The incidence of STEMI gradually decreased (from 90.97% to 79.51%) with the increase of age, while the incidence of NSTEMI showed a trend to increase (from 9.03% to 20.49%). As age increases, the incidence of LAD artery occlusion decreased from 60.26% to 52.01%, while the incidence of right coronary artery occlusion increased from 28.51% to 38.95%. The prevalence of circumflex artery and left main artery occlusion did not change with age.

**Table 2:** Characteristics of patients with AMI grouped by age

	<40 years	40-49 years	50-59 years	60-69 years	70-79 years	≥80 years	*P* value
**No. of patients**	2315	8444	15046	17263	14717	6428	–
**Types of AMI, n (%)**							
STEMI	2106 (90.97)	7523 (89.09)	13030 (86.60)	14810 (85.79)	12086 (82.12)	5111 (79.51)	<0.001
NSTEMI	209 (9.03)	921 (10.91)	2016 (13.40)	2453 (14.21)	2631 (17.88)	1317 (20.49)	<0.001
**Lesion location, n (%)**							
Anterior wall	1116 (48.21)	3804 (45.05)	6344 (42.16)	7253 (42.01)	5796 (39.38)	2474 (38.49)	<0.001
Inferior wall	774 (33.43)	2946 (34.89)	5354 (35.58)	5818 (33.70)	4792 (32.56)	1935 (30.10)	<0.001
Others	425 (18.36)	1694 (20.06)	3348 (22.25)	4192 (24.28)	4129 (28.06)	2019 (31.41)	<0.001
**Affected vessels, n (%)**							
LAD	1395 (60.26)	4806 (56.92)	8162 (54.25)	9608 (55.66)	7812 (53.08)	3343 (52.01)	<0.001
RCA	660 (28.51)	2647 (31.35)	5029 (33.42)	5909 (34.23)	5459 (37.09)	2504 (38.95)	<0.001
LCX	260 (11.23)	991 (11.74)	1855 (12.33)	1746 (10.11)	1446 (9.83)	581 (9.04)	0.12
**Coronary occlusion, n (%)**	1396 (60.30)	4883 (57.83)	8459 (56.22)	9270 (53.70)	8175 (55.55)	3697 (57.51)	0.22
**PCI-target vessels, n (%)**							
Single vessel	2183 (94.30)	7607 (90.09)	13462 (89.47)	15305 (88.66)	12961 (88.07)	5866 (91.26)	0.01
Multi-vessels	132 (5.70)	837 (9.91)	1584 (10.53)	1958 (11.34)	1756 (11.93)	562 (8.74)	0.01

LAD, left anterior descending; LCX, left circumflex; RCA, right coronary artery; see other abbreviations in Table 1.

### Management of AMI

**Figure 1:**
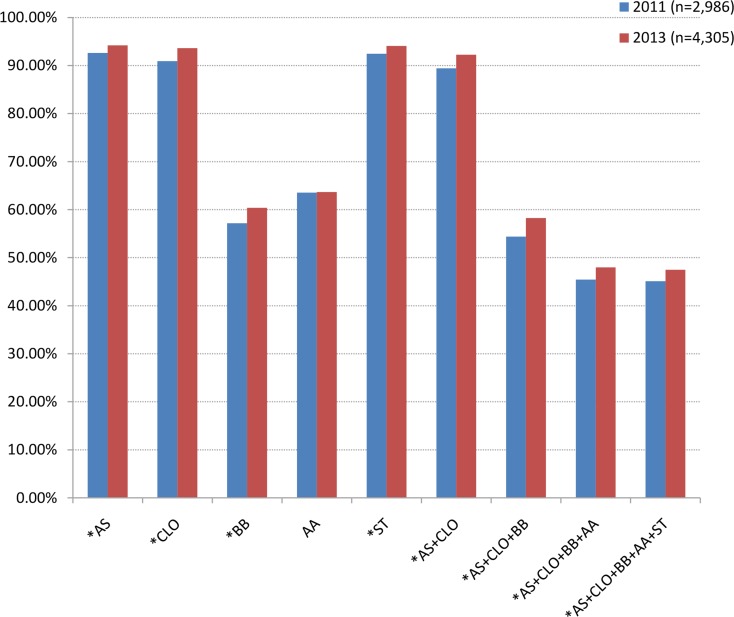
Percentage of drug use during hospitalization *: *P* < 0.05 compared with 2011. AA, ACEI/ARB; AS, aspirin; BB, β-blocker; CLO, clopidogrel; ST, statin.

A total of 7,291 patients’ records were available for evaluating medical strategies. More than 90% of patients had received aspirin, clopidogrel and statin treatment, while approximately half of patients were given β-blockers and/or ACEI/ARB. Compared to the year 2011, the use of all the above evidence-based agents in 2013 increased except the ACEI/ARB (Figure 1).

**Figure 2:**
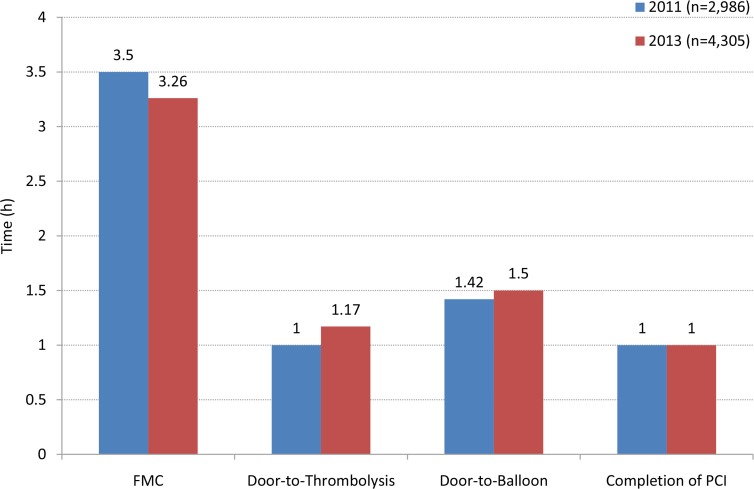
Management efficacy for AMI

The approach of patient administration was analyzed to understand the transportation mechanism, treatment options and social burden. For patient transportation, the rates of using ambulances and taxis/private cars were 39.89% and 60.11% in 2011 and 39.93% and 60.07% in 2013, respectively (*P* = 0.98). In year 2011 and 2013, the emergency management measures did not differ significantly. PCI was the most commonly performed measure (46.12% and 43.39%), followed by the conservative therapy (42.06% and 43.16%), only 11.82% and 13.45% of patients underwent emergency thrombolysis. For the time point of intervention, there was no significant shortening in onset-to-FMC time (median, 3.5h *vs*. 3.26h), door-to-thrombolysis time (median, 1.00h *vs*. 1.17h), door-to-balloon time (median, 1.42h *vs*. 1.5h), and the time for completion of PCI (median, 1.00h *vs*. 1.00h) from 2011 to 2013 (Figure 2). The recanalization rate of PCI in year 2013 was, however, significantly improved compared to year 2011 (Table 3).

**Table 3:** General information on AMI management in the year 2011 and 2013

	2011 (n=2,986)	2013 (n=4,305)	*P* value
**Transportation, n (%)**			0.981
Ambulances	1191 (39.89)	1719 (39.93)	-
Taxis/private cars	1795 (60.11)	2586 (60.07)	-
**Recanalization rate, fraction (%)**			
Emergency thrombolysis	651/675 (96.44)	1151/1211 (95.05)	0.165
Emergency PCI	3203/3336 (96.01)	6557/6648 (98.63)	<0.001
**Hospital stay, days**	10.0 (7.0-14.0)	9.0 (6.0-13.0)	<0.01
**Total costs, yuan**	36939.2 (13089.3-55123.7)	37798.8 (14858.9-64754.6)	0.09
**Payment, n (%)**			
Paid by medical insurance	1818 (60.88)	2993 (69.52)	<0.001
Patients’ paid expense	607 (20.33)	601 (13.96)	<0.001
**Outcome, n (%)**			
Improved	2678 (89.69)	3928 (91.24)	0.027
No response	21 (0.70)	21 (0.49)	0.271
Death	135 (4.52)	153 (3.55)	0.038
**Post-discharge whereabouts, n (%)**			0.574
Going home	2687 (89.99)	3892 (90.41)	-
Going to rehabilitation facility	299 (10.01)	413 (9.59)	-

PCI, percutaneous coronary intervention.

The main reasons for not implementing primary PCI are as follows: hospitals do not have catheterization laboratory (declined from 50.32% to 23.13%); patient factors (in the order of frequency), including the cost of PCI (declined from 37.79% to 18.74%), requirement of patient/relatives’ consent and health insurance coverage.

Compared to year 2011, patients with AMI had shorter hospital stay (median, 9 *vs*. 10 days), without changes in the total medical cost (median, ¥36939.2 *vs*. ¥37798.8) in 2013. There was a notable change in the structure of payment, with an increased payment from the health insurance (from 60.88% to 69.52%) and a reduced payment from the patients (from 20.33% to 13.96%). There were less in-hospital deaths in 2013 than in 2011 (3.55% *vs*. 4.52%, *P* = 0.038). The majority of patients chose to go home after discharge (89.99% in 2011 *vs*. 90.41% in 2013) other than going to rehabilitation facility for further therapy (10.01% in 2011 *vs*. 9.59% in 2013, Table 3).

### Quality control indicators

**Figure 3:**
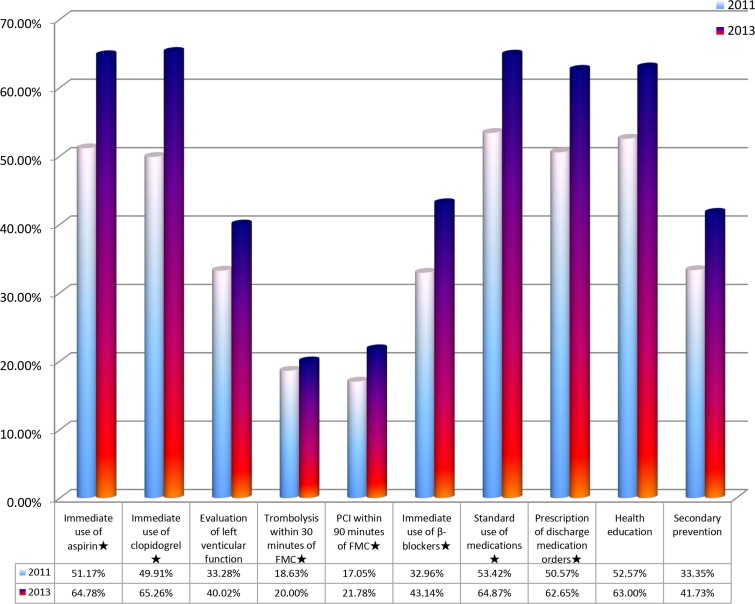
Completion rate of quality control indicators in year 2011 and 2013 *: the core indicators.

The quality control indicators of AMI [4] are as follows: 1. Immediate use of aspirin or clopidogrel after admission if no contraindication (Class I Grade a); 2. Evaluation of left ventricular function (Class I Grade b); 3. Thrombolysis within 30 minutes of the FMC (Class I Grade a) if no contraindication; 4. PCI within 90 minutes of the FMC if no contraindication (Class I Grade a); 5. Immediate use of β-blockers after admission if no contraindication (Class I Grade a); 6. Standardized application of medications during hospitalization, including β-blocker, aspirin/clopidogrel, ACEI/ARB, and statins if no contraindication (Class I Grade b); 7. Prescription of discharge medication, including β-blocker, aspirin /clopidogrel, ACEI /ARB and statins if no contraindication (Class I grade b); 8. Evaluation and management of blood lipid levels (Class I Grade b); 9. Quit smoking, health education, and secondary prevention education. In these indicators, the indicators 1, 6, 7 and 9 had a completion rate over 50%. The mean completion rate for all quality control indicators in 2013 was higher than that in 2011 (48.72% *vs*. 39.32%, *P* < 0.01), with exception (no difference) for thrombolysis within 30 minutes of the FMC (Figure 3).

## DISCUSSION

The incidence of AMI in China shows a trend of increase, but no report has described the present characteristics and management improvement of AMI in Chinese population. In this study, we analyzed the data of AMI patients in tertiary general hospitals from SDQCS database, and found that from 2011 to 2013, significant improvements have been achieved in recanalization rate of PCI and in-hospital mortality of AMI, with no increase in medical cost. As a limitation of the registration system, AMI cases in small hospitals have not been registered to the system. However, the data are still representative because the majority of patients with AMI are treated in the tertiary general hospitals while most of AMI patients who initially entered small hospitals were transported to the tertiary general hospitals for recanalization therapy to meet the Chinese guideline.

Different from a large proportion of NSTEMI reported in the previous surveys in Western countries [5–7], in the present study, STEMI accounted for the majority of AMI. This may be partly associated with the relatively larger smoking population in China [8]. It has been reported that cigarette consumption is an important risk factor for STEMI compared with NSTEMI [9], and almost 50% of STEMI can be attributable to smoking [10]. Similarly, STEMI was observed in nearly 79% of total cohort in a national registry from Japan [11], suggesting that other factors such as ethnicity and lifestyle may also contribute to the aforementioned difference in AMI type. Similar to the reports by others [12–14], we confirmed that STEMI mainly affects the anterior wall and the inferior wall in Chinese population with culprit vessels most affected in the LAD artery and the RCA. Patients with NSTEMI also most commonly occurred in LAD with less operations of emergency PCI.

Although the quality of medical care, such as the recanalization rate of PCI and in-hospital mortality has improved in the past 3 years, the onset-to-FMC time is still longer in China (3.3-3.5 h) than in Western countries in which the median onset-to-FMC time is 2 to 3 hours [15, 16], resulting in a consequent delay of thrombolysis and PCI. Thus, more efforts should devote to fill the gaps since timely reperfusion therapy, especially successful implementation of emergency PCI, are associated with better outcomes in patients with AMI [17]. In addition to the geodemographic factors and community emergency medical care system, the patient education should also be strengthened. Most of patients were transferred to hospitals by private vehicles instead of ambulance, which has been associated with pre-hospital delay [18] and may lead to an increased probability of cardiac arrest [19]. It deserves more attention for the health care professionals to provide better education to the public, especially those at high risk of MI, to help them recognize the warning symptoms and survival strategies. Furthermore, consistent with several investigations about Chinese population [20–22], the time of door-to-thrombolysis was evidently longer in the present study than that recommended by guidelines. The main reason for the in-hospital delay of reperfusion therapy is due to the delay in consent provision, which is a persistent problem for the prolonged door-to-thrombolysis time [22, 23].

Patients who underwent PCI showed better outcomes than those receiving thrombolysis and drug treatment. It should be noted that in our study, a recanalization rate of thrombolysis is over 95%, which is higher than previously reported. This may be attributed to that most of thrombolytic therapies were strictly implemented to the ideal candidates as guideline-recommended (within 3 hours of onset in China) [24]. The rate of reperfusion therapies in the present study was comparable with that documented in the China PEACE-Retrospective Acute Myocardial Infarction Study [25] but lower than those reported in Europe and USA [26–28].

The use of medicine has basically met the evidence-based standards for AMI treatment, including aspirin, clopidogrel, ACEI/ARB, β-blockers and statins, but the rate of using ACEI/ARB and β-blockers was lower than other medications. It is presumable that physicians were cautious about the prescription of ACEI/ARB and β-blockers due to their potential adverse effects on blood pressure and the negative inotropic effect. Although more drugs and devices were utilized, we found no increase in the total medical cost. This is attributed to the shortened time of hospitalization. The proportion of patients’ paid expense is significantly decreased due to the expended coverage from the government provided medical insurance.

The completion rate of quality control indicators remains to be improved, although major progress has been made in the medical management, including the immediate use of aspirin/clopidogrel, increased coverage of social insurance, standardized use of drugs, and timely implementation of reperfusion therapy following FMC, etc. Further efforts should focus on strengthening of health care education to the public to enrich public knowledge of AMI, improvement of patient transportation system and compliance to the guideline-directed management.

## CONCLUSIONS

Compared with the Western countries, STEMI accounted for the majority of AMI, and the AMI management in China basically meets the standards of the quality control of guidelines. With the improvement of management, there was no increase in the total medical cost, while the patient's paid portion was actually reduced. In future, improvement of transportation strategy and the public medical education are recommended to shorten the onset-to-FMC time to further improve the outcome of AMI patients.

## MATERIALS AND METHODS

### Study population

Since 2009, the China Hospital Association has implemented the Single Disease Quality Control System (SDQCS), a source that includes details on disease management at general hospitals in China. To ensure the quality of the data reported to SDQCS, a standardized disease-specific reporting approach was set up for data reporting and monitoring. From the SDQCS, we identified admissions from 492 tertiary general hospitals located in all of the 32 provinces of China with a primary diagnosis of AMI in 2011 and 2013 according to the International Classification of Diseases, Version 10 (ICD-10). ICD-10 codes I21.0 to I21.3 were classified as ST-segment elevation myocardial infarction (STEMI); and ICD-10 code I21.4 was classified as non-ST segment elevation myocardial infarction (NSTEMI). Because the Chinese Guideline for Percutaneous Coronary Intervention [29] was released in 2012, we used the data in 2011 and 2013 to evaluate whether guideline-based treatment improves the outcomes of AMI patients. The study was approved by the ethics committee of Zhongnan Hospital of Wuhan University, Wuhan, China.

### Evaluated indicators

The following indicators were evaluated: 1) demographic characteristics: age; 2) baseline information before and after admission: ways of admission, the time from onset to first medical contact (FMC), FMC to thrombolysis, FMC to balloon and the duration of PCI; 3) general treatment information: thrombolysis, emergency PCI, conservative treatment; 4) recanalization rate; 5) management after discharge: medications prescribed, health education; and 6) economic burden: days of hospital stay, medical expenses. The recanalization of culprit vessel for thrombolytic therapy was defined when meeting a ≥ 50% resolution of ST-segment elevation within 60 to 90 minutes and significant relief of chest pain within 2 hours. The recanalization following percutaneous coronary intervention (PCI) was manifested by a TIMI flow grade 2 or 3 of the infarcted coronary artery. FMC was defined as the point of arrival of medical personnel to the patient or patient enters emergency room if the patient is self-transported.

### Statistic analysis

Continuous data were expressed as mean±standard deviation and median (interquartile range), respectively. The categorical variables were presented as numbers (percentage). The difference between 2 groups was assessed by Student's *t*-test, Mann-Whitney U test or Chi-square test, when appropriate. The trends of variables across different age groups were detected by the linear-by-linear Chi-square test. Statistical analysis was performed using Empower States (www.empowerstats.com, X & Y Solution, Inc Boston MA.) and R software (http://www.R-project.org). A *P* value < 0.05 was considered as significant.

1
